# sRNAbench and sRNAtoolbox 2022 update: accurate miRNA and sncRNA profiling for model and non-model organisms

**DOI:** 10.1093/nar/gkac363

**Published:** 2022-05-12

**Authors:** Ernesto Aparicio-Puerta, Cristina Gómez-Martín, Stavros Giannoukakos, José María Medina, Chantal Scheepbouwer, Adrián García-Moreno, Pedro Carmona-Saez, Bastian Fromm, Michiel Pegtel, Andreas Keller, Juan Antonio Marchal, Michael Hackenberg

**Affiliations:** Chair for Clinical Bioinformatics, Saarland University, 66123 Saarbrücken, Germany; Department of Pathology, Cancer Center Amsterdam, Amsterdam UMC, VU University, Amsterdam 1081HV, Netherlands; Genetics Department, Faculty of Science, Universidad de Granada, Campus de Fuentenueva s/n, 18071 Granada, Spain; Bioinformatics Laboratory, Biomedical Research Centre (CIBM), PTS, Avda. del Conocimiento s/n, 18100 Granada, Spain; Excellence Research Unit “Modeling Nature” (MNat), University of Granada, Spain; Genetics Department, Faculty of Science, Universidad de Granada, Campus de Fuentenueva s/n, 18071 Granada, Spain; Bioinformatics Laboratory, Biomedical Research Centre (CIBM), PTS, Avda. del Conocimiento s/n, 18100 Granada, Spain; Excellence Research Unit “Modeling Nature” (MNat), University of Granada, Spain; Department of Pathology, Cancer Center Amsterdam, Amsterdam UMC, VU University, Amsterdam 1081HV, Netherlands; Department of Neurosurgery, Cancer Center Amsterdam, Amsterdam UMC, VU University, Amsterdam 1081HV, Netherlands; Centre for Genomics and Oncological Research. GENYO. Pfizer / University of Granada,18016 Granada, Spain; Centre for Genomics and Oncological Research. GENYO. Pfizer / University of Granada,18016 Granada, Spain; The Arctic University Museum of Norway, 9006 Tromso, Norway; Department of Pathology, Cancer Center Amsterdam, Amsterdam UMC, VU University, Amsterdam 1081HV, Netherlands; Chair for Clinical Bioinformatics, Saarland University, 66123 Saarbrücken, Germany; Excellence Research Unit “Modeling Nature” (MNat), University of Granada, Spain; Department of Human Anatomy and Embryology, Institute of Biopathology and Regenerative Medicine, University of Granada, 18011 Granada, Spain; Instituto de Investigación Biosanitaria ibs.GRANADA, University Hospitals of Granada-University of Granada, Spain; Conocimiento s/n 18100, Granada. Spain; Genetics Department, Faculty of Science, Universidad de Granada, Campus de Fuentenueva s/n, 18071 Granada, Spain; Bioinformatics Laboratory, Biomedical Research Centre (CIBM), PTS, Avda. del Conocimiento s/n, 18100 Granada, Spain; Excellence Research Unit “Modeling Nature” (MNat), University of Granada, Spain; Instituto de Investigación Biosanitaria ibs.GRANADA, University Hospitals of Granada-University of Granada, Spain; Conocimiento s/n 18100, Granada. Spain

## Abstract

The NCBI Sequence Read Archive currently hosts microRNA sequencing data for over 800 different species, evidencing the existence of a broad taxonomic distribution in the field of small RNA research. Simultaneously, the number of samples per miRNA-seq study continues to increase resulting in a vast amount of data that requires accurate, fast and user-friendly analysis methods. Since the previous release of sRNAtoolbox in 2019, 55 000 sRNAbench jobs have been submitted which has motivated many improvements in its usability and the scope of the underlying annotation database. With this update, users can upload an unlimited number of samples or import them from Google Drive, Dropbox or URLs. Micro- and small RNA profiling can now be carried out using high-confidence Metazoan and plant specific databases, MirGeneDB and PmiREN respectively, together with genome assemblies and libraries from 441 Ensembl species. The new results page includes straightforward sample annotation to allow downstream differential expression analysis with sRNAde. Unassigned reads can also be explored by means of a new tool that performs mapping to microbial references, which can reveal contamination events or biologically meaningful findings as we describe in the example. sRNAtoolbox is available at: https://arn.ugr.es/srnatoolbox/.

## Graphical Abstract

**Graphical Abstract:**
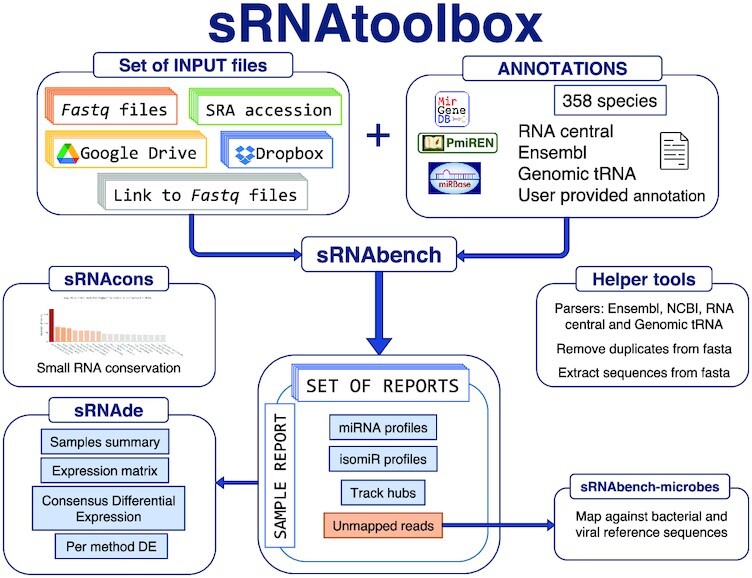
Overview of sRNAtoolbox improvements and current core features. Updated annotations for accurate miRNA and small RNA expression profiling and downstream analysis in over 300 species.

## INTRODUCTION

**Figure 1.**
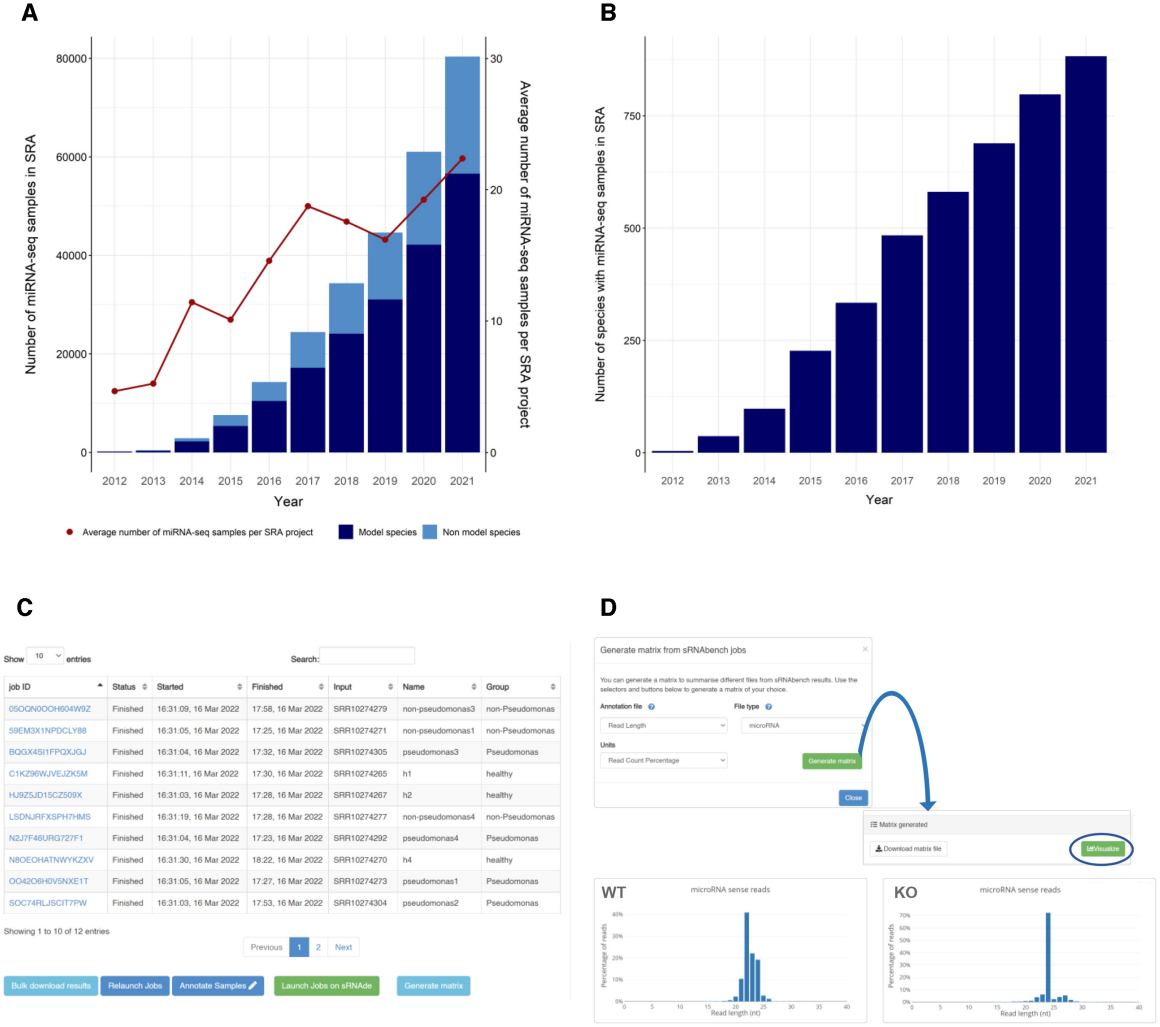
Evolution of miRNA-seq data hosted on SRA and consequent improvements in sRNAbench. (**A**) Accumulated number of samples in SRA per year and the increasing tendency of samples per project. (**B**) Number of species with publicly available miRNA-seq data on SRA. Both graphics have been generated after querying the database (https://www.ncbi.nlm.nih.gov/sra/) with ‘Library selection’ field set to miRNA-seq. (**C**) New sRNAbench status page that allows for on-the-go annotation and direct launching of differential expression jobs. (**D**) New helper tool that can summarize and visualize statistics and count matrix. The microRNA read length distributions were obtained for two parental cell lines (WT; SRR3174960, SRR3174961) and two DICER KO cell lines (KO: SRR3174967,SRR3174968) showing clear differences between these two conditions.

MiRNA sequencing (or small RNA sequencing) is a well-established method to quantify, detect and annotate microRNAs (miRNAs) as well as other small non-coding RNAs, based on Next Generation Sequencing (NGS) technologies (1). Current metadata based on both publicly available miRNA-seq samples in the Sequence Read Archive (SRA) (2) and miRNA publications available on Pubmed (3) show that the research field of small non-coding RNAs is actively gaining ground in both model and non-model organisms (see Figure 1A, B). Given the strong need for miRNA-seq analysis, the Bioinformatics community developed many different web tools and pipelines that can perform, at the very least, a straightforward quantification analysis of microRNAs. A short list includes miRDeep (4), miRMaster (5), miRge (6), SeqBuster (7) and sRNAbench (8,9), which succeeded miRanalyzer (10), although many more have been developed (5). Beyond quantification, other types of analysis deal with miRNA pathway and target detection (11,12), quality control of sequencing data (13,14) or more specific aspects like arm-shifting or miRNA sponges (15,16). In parallel to software developments, different projects have recently attempted to improve and curate microRNA annotations by removing false positive entries, a known source of bias in downstream results (17). The metazoan specific MirGeneDB2.1 (18) and plant specific PmiRen2.0 (19) are therefore recommended for the correct profiling of *bona fide* miRNAs using sRNAbench.

Most of the tools mentioned above focus on a small subset of species (5) making the access to a broad scope of miRNA analysis particularly difficult for researchers working in non-model species. In contrast, sRNAbench currently provides miRNA profiling for 358 different species through MirGeneDB2.1, PmiRen2.0 and miRBase (release 22.1) (20), covering a broad taxonomic spectrum. We find that using precise up-to-date annotations is one of the crucial aspects needed to provide a reliable quantification analysis of miRNA-seq data. Additionally, sRNAtoolbox also includes Ensembl (21) genomic annotations and assemblies from 441 organisms. Furthermore, RNAcentral (22) was parsed and classified for each species, allowing for small RNA quantification even for species without microRNA complement annotation. Due to the flexible design of sRNAbench, users can select custom combinations of microRNA complements which is especially useful when no microRNA annotations are available for the species of interest. Finally, bacterial and viral genomic annotations have also been compiled from NCBI (23) to check for contamination in miRNA-seq libraries, a common problem that is frequently overlooked.

Together with the updated annotations, the current release provides a wide variety of library preparation protocols that can be preprocessed as well as an algorithm to automatically detect the protocol from a fraction of the miRNA-seq reads. This is useful to assist users who are unsure of what library preparation protocol was used for their data of interest, not an uncommon event in samples available in SRA. Additional improvements designed to increase the usability of sRNAbench include data import from SRA, Google Drive and Dropbox as well as on-the-go annotation of samples for downstream differential expression analysis with sRNAde.

In summary, the current update aims to benefit an even broader range of miRNA researchers, including those working on organisms traditionally left out such as plants and non-model organisms. Moreover, we focused on providing fast and versatile analysis that rely on accurate up-to-date annotations. Above-mentioned improvements as well as further additions are discussed in the next sections of this article.

## IMPROVEMENTS AND CURRENT FEATURES

### Reference annotations

The current sRNAtoolbox version relies on updated miRNA, ncRNA and genomic annotations, making it a substantial upgrade from the previous release (see Table 1 for a comparison to the 2019 release (8)). sRNAtoolbox now includes MiRGeneDB2.1, PmiRen2.0 and miRBase (release 22.1) for microRNA profiling which is still the most common application and the center piece of this web server. The authors strongly recommend the usage of MiRGeneDB and PmiREN given the remarkable curation carried out in these databases to remove false positive entries. Other small non coding annotations available include, in the order used by sRNAbench: GtRNAdb (24) for tRNA, RNAcentral (release 20) (22), ncRNA (from Ensembl) and cDNA (from Ensembl) (21). Genomic assemblies and annotations were updated to Ensembl 104 (Ensembl Genomes 51 for species belonging to EnsemblMetazoa or EnsemblPlants) bringing the total count of plant and animal genomes hosted in our database to 441. Furthermore, we have also compiled a wide spectrum of genomic indexes from the NCBI Microbial Genomes resource (25) since mappings to bacterial, viral or even fungal genomes can be used to trace contamination issues as well as to analyze and reveal pathogen/host interactions. Indexing the entire NCBI Microbial Genomes database would be unfeasible, therefore, we generated 30 different reference files, one per phylum, where each species is represented by one genome. In total, nearly 10 000 bacterial species have been added. Virus sequences obtained from NCBI virus (25) have been treated in a similar way obtaining over 10000 sequences classified by host organism, i.e. human, vertebrates, invertebrates, plants, land-plants, etc. Those libraries are available in our new tool sRNAbench-microbes.

**Table 1.** A comparison between sRNAtoolbox 2022 and 2019 databases

	sRNAtoolbox 2022	sRNAtoolbox 2019
*Animal genomes*	Ensembl 104 (or 51 for Metazoan), 350 genomes	Ensembl v91, 97 genomes
*Plant genomes*	Ensembl Plants v51, 91 genomes	Ensembl Plants, 48 genomes
*Bacterial collection*	NCBI Microbial Genomes (one genome per genus), 3004 genomes	NCBI Microbial Genomes (one genome per genus), 781 genomes
*Bacterial genomes*	NCBI Microbial Genomes (one genome per species), 9655 genomes	Not available
*Virus genomes*	NCBI virus (one genome per species), 10301 genomes	Not available
*RNA annotations*	RNA central release 20 (snoRNA, snRNA,…)	RNA central release 13
*miRNA annotations*	pMiren, MirGeneDB, miRbase	MirGeneDB, miRbase
*Number of different species with miRNA complement*	358	293

Finally, we also generated a more condensed collection of bacterial genomes with one representative species per genus, which is available for profiling in sRNAbench (genome mode) in order to obtain first clues about the existence of putative contamination.

### sRNAbench

As mentioned above, sRNAbench (26) is the most frequently used tool in sRNAtoolbox and as such, most improvements were centered on it. From sRNAbench usage statistics and SRA metadata (see Figure 1A) it became obvious that most users are interested in simultaneous analysis of several samples. This is why, in our previous release, we already made it possible to launch multiple sRNAbench jobs from just one tool (batch mode), which has been updated to include dataset import from SRA, URLs or cloud file storage as well as regular file upload. The most common downstream analysis is differential expression which requires sample annotation, i.e. at least two different conditions like healthy/cancer need to be specified for the analyzed samples. This assignment can now be easily performed by annotation files in Excel or plain text tabular format. Annotated samples can then be directly analyzed with sRNAde from the job status page of sRNAbench. Besides microRNA profiles, sRNAbench generates a high number of other results such as relative read abundance as a function of RNA species. We added now a helper tool, accessible through the ‘Generate Matrix’ button at the sRNAbench status page that allows download and visualization of this information (see Figure 1C). An interface guides the user through the available features that can be summarized (Figure 1D).

#### Automated protocol detection

Although detection of unknown adapters was already possible in previous releases of sRNAbench, we have introduced an algorithm that allows the automatic detection of any library preparation protocol, including those based on random adapters. In short terms, the method works as follows:

1. Check if input is adapter-trimmed (different read lengths in the input file).
2. Test if any of the most used commercial adapters (Illumina TrueSeq™, NEBnext™, Bioo Scientific Nextflex™, Clontech SMARTer™, Qiaseq™) can be detected in >50% of all reads. Additionally, at least 20% of the microRNA complement of the species needs to be covered by those reads.
3. If the strict requirements in the first step are not met, either because the correct adapter was not tested or the sample is of low quality, the previously introduced ‘Guess Adapter’ function is used. Among all tested adapters, the most frequent one is chosen to test for the existence of random sequences.
  1. Detect canonical miRNA sequences within the adapter trimmed reads and calculate the lengths of the flanking sequences.
  2. Infer the most likely length of random nucleotides from this information.

#### Filter of low complexity reads

By default, low complexity reads are now filtered out as they generally map to a high number of loci which unnecessarily increases the computation time. The complexity ratio is calculated as the fraction of the most frequent nucleotide. By default, all reads are removed if 80% or more of all residues correspond to only one nucleotide.

### sRNAde

Differential expression analysis can now be launched directly from the sRNAbench job status page. Furthermore, samples can be annotated and reannotated on the go by means of spreadsheets or tabular files. This feature allows for several differential expression analyses to be easily launched from the same page reassigning or regrouping the samples into different conditions or leaving out outlier samples.

In summary, sRNAde provides consensus and individual differential expression results of miRNA expression calculated by five different methods (edgeR (27),DESeq (28), DESeq2 (29), NOISeq (30) and Student's *t*-test (31)). It also provides several statistics and visualizations concerning sRNA mapping distributions and preprocessing to help users acquire an appropriate overview of their project as well as to identify potential issues and outliers in their dataset.

### sRNAblast

This tool performs blast searches with user-provided small RNA reads, typically unmapped or unidentified reads from sRNAbench jobs. Small sequences can be mapped with the same quality to many different database entries, either because they are conserved or by chance alone. From the raw blast output, only matches with the highest score above the identity threshold (90%) are kept for further analysis. In the previous version, all these matches were presented to the users making it considerably difficult to decide whether those results actually indicate the presence of genetic material from the detected species or are mere false positive mappings.

In order to provide a more meaningful output, we first determine a list of species ordered by the overall number of assigned reads reasoning that the most likely origin is from the species with the highest overall number of mappings.

Each read with several equal blast matches is then assigned to the species with the highest number of total matches. Consequently, for each input read only one hit is presented on the output page.

### miRNAconsTarget

The purpose of miRNAconsTarget is to determine the consensus mRNA target from several miRNA/mRNA target prediction tools. In previous versions, this consensus was defined by pairs of miRNA/mRNA regardless of the position of the predicted interactions inside the target sequence. Besides this consensus, the current update also includes the positional consensus for both animal and plant predictions. With this restriction, a target is only considered consensus if all chosen methods predict an overlapping interaction in the same region. Three new output files are reported:

- **positionalConsensus:** the miRNA/mRNA interactions with spatial intersection of all selected methods and their target sequence coordinates
- **multipleTargets:** a list of mRNAs that have more than one target for a specific miRNA
- **perTranscript:** a list of all miRNA targets per target sequence

### NEW TOOLS

#### sRNAcons

This new tool is intended to provide an estimation of the conservation depth of small RNAs. User provided sRNA sequences in fasta format are mapped against all animal or plant assemblies available in sRNAtoolbox. The alignment is performed using Bowtie1 (32) with -v option (the full sequence will be aligned) allowing for 0, 1 and 2 mismatches. Two output files are generated: (i) the conservation depth for all input sequences, i.e the percentage of genomes in which the sequence was found and (ii) the percentage of mapped input sequences per genome. We provide a working example with a discussion on how to interpret these figures. To avoid overestimation of duplicated input sequences (which can easily happen in miRNA annotations as frequently more than one gene exists for a functional mature sequence), a non-redundant set of input sequences is first generated by randomly assigning only one fasta ID. The tool provides a downloadable report on the detected redundancies.

#### sRNAbench-microbes

Contamination of small RNA libraries is an important issue, particularly in low-input samples. In such cases, contamination can easily reach similar concentrations to those of the starting material, which can lead to high percentages of reads not mapping to the assembly or libraries of interest. To detect reads potentially originating from bacterial of viral contamination, we branched the original sRNAbench into a new tool that aligns unmapped reads from previously finished sRNAbench jobs to several collections of bacterial and viral reference sequences generated in the fashion described above. The tool reports summary results like the relative abundance found for each phylum or the detailed quantification for each sequence contained in the reference libraries. Two parameters need to be provided: the number of allowed mismatches and the sRNAbench job ID. The tool aligns the unmapped reads of the sRNAbench job to all 30 bacterial phylum reference sequences and 12 virus reference data sets using sRNAbench default.

## WORKING EXAMPLES

**Figure 2.**
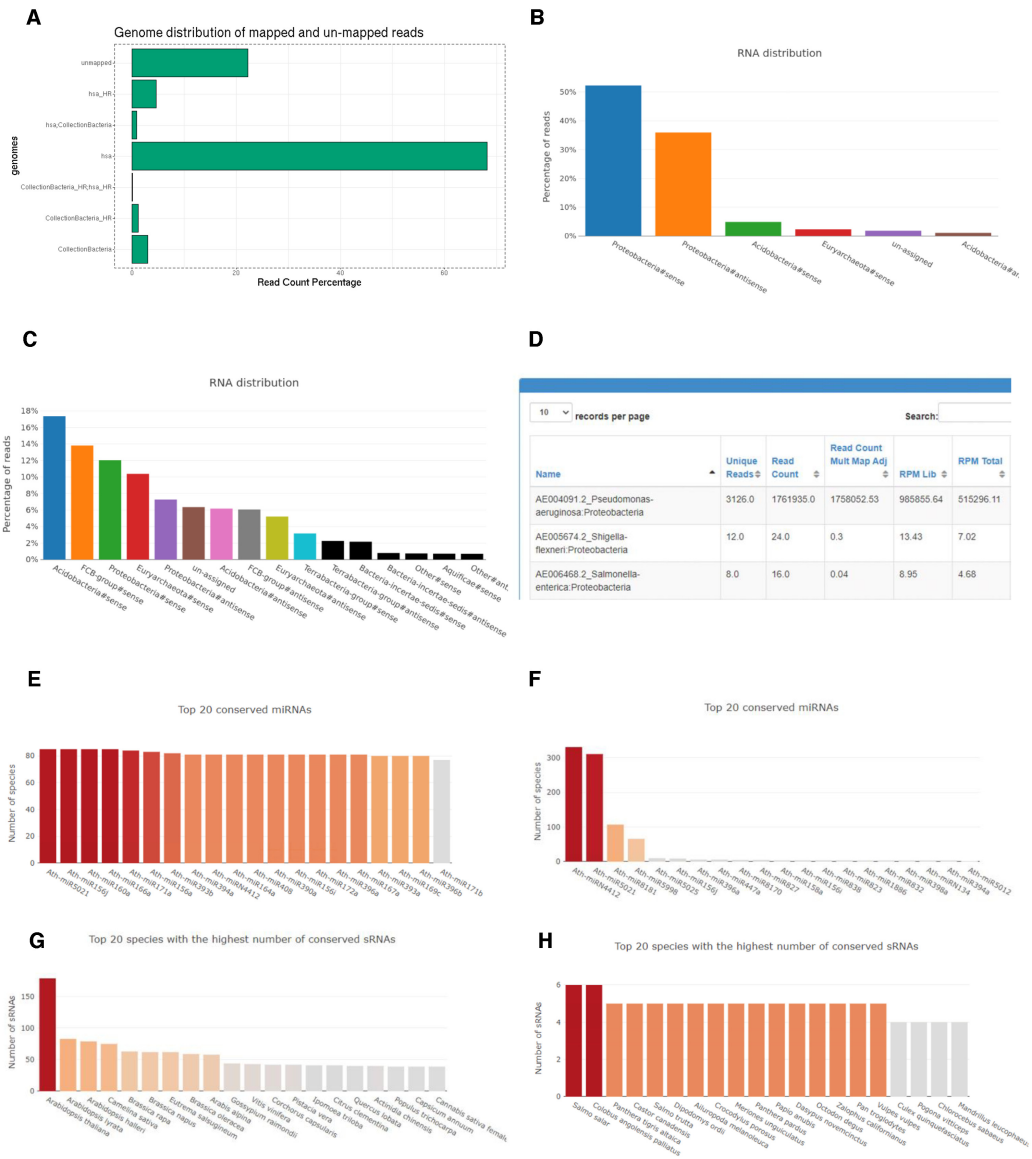
(**A**) Genome distribution (human and bacterial collection) of PA + sample SRR10274305, (**B**) relative frequencies of reads mapped to different phylum reference libraries (SRR10274305), (**C**) relative frequencies of reads mapped to different phylum reference libraries of a healthy subject (SRR10274270), (**D**) results table of reads mapped in sense direction to proteobacteria reference sequences, (**E**) graphical representation of the 20 most conserved Arabidopsis thaliana PmiREN microRNAs, (**F**) plant species that contain most exact Arabidopsis thaliana miRNA sequences, (**G**) 20 miRNAs with most matches to animal genomes and (**H**) animal genomes with most exact matches of A. thaliana miRNA sequences.

In order to illustrate the usefulness of the introduced improvements, we used small RNA sequencing data from the SRA project SRP225537. This study aims to reveal miRNA implication in Bronchiectasis (33). To achieve this, the authors sequenced small RNAs from sputum extracted exosomes from three conditions: (i) healthy subjects, (ii) non-Pseudomona colonization (PA–) and (iii) Pseudomona colonization (PA+). The colonization with *Pseudomonas aeruginosa* (PA) was determined by at least two positive cultures. We first reanalyzed 12 samples (4 from each group) by means of sRNAbench including the bacterial collection. Figure 2A shows the results for the PA+ sample SRR10274305. Most reads can be mapped to the human genome but over 20% are unmapped and around 3% of all reads map to the bacterial collection (one bacterial genome per genus). In order to explore in more detail the unmapped reads and those of putative bacterial origin, we first re-launched all 12 jobs without using bacterial collection and then submitted the unmapped reads to sRNAbench-microbes. Figure 2B shows the phylum distribution of SRR10274305 reads. Two interesting facts can be observed: first, virtually all previously unmapped reads can now be assigned (only 2.1% of these remain unmapped) and second, nearly all reads are mapped either in sense or antisense direction to the proteobacteria phylum to which *Pseudomonas aeruginosa* belongs. Interestingly, this holds for all PA+ samples while healthy controls do not show high abundance of Proteobacteria (Figure 2C for healthy sample SRR10274270). This suggests that the presence of PA can be detected in sputum exosome small RNA samples, providing evidence that this tool can detect biologically meaningful genetic material from bacterial/viral origin. Finally, the detailed output page reveals that indeed nearly all sense mappings to proteobacteria phylum sequences (Figure 2B) belong to *P. aeruginosa* (AE004091.2) (Figure 2D).

Next we will briefly discuss possible applications of the sRNAcons tool. In its current version, this tool merely maps the input reads to all animal or plant genomes hosted in sRNAtoolbox. If the input reads are very short, false positive mappings can be expected, i.e. matches to genomes in which no homologous sequence exists. Since it's a general purpose tool, no special treatment is given to microRNA input reads as would be taking into account the known conservation of the seed regions (a likely future improvement). One possible application could be to test the conservation depth of a miRNA complement for a new species or novel microRNAs. Depending on the input, high conservation can have different explanations. For metazoan, strongly conserved microRNAs are supposed to be already known for quite some time (34) and high conservation of putatively novel miRNAs could be as well due to the overlap with other, conserved genomic elements. Many of such novel miRNAs might actually be false positives that are in fact fragments of highly conserved sequences such as ribosomal or small nucleolar RNAs.

To illustrate the usage of sRNAcons, we analyzed the *Arabidopsis thaliana* miRNA complement from PmiREN2.0 without allowing any mismatches. Figure 2E shows the 20 most conserved microRNAs when mapping against all plant genomes. Not surprisingly, well known families like miR156 and miR166 are among the most highly conserved sequences. Figure 2G shows the species that contain most sequences of the miRNA complement. The order of the species reflects the known taxonomy: first other species from the Arabidopsis genus followed by species from the Brassicaceae family. In theory, no homologous microRNAs exist between plants and metazoan, therefore we repeated the analysis with all animal genomes as negative control. Figure 2F, however, shows that at least two highly conserved sequences, Ath-miRN4412 and Ath-miR5021, perfectly map to 331 and 311 animal genomes, respectively. Both microRNAs are located within protein coding genes (AT4G39840, AT1G04440) and therefore the conservation beyond the plant kingdom is due to the function of these highly conserved genes and not to the microRNA function. Finally, Figure 2H shows the distribution of mappings in animal species, in clear contrast to Figure 2G. The highest number of matches is 6 in two completely unrelated species, *Colobus angolensis palliatus* and *Salmo salar*. Two of these matches are caused by Ath-miRN4412 and Ath-miR5021 but the rest are likely due to chance alone. This shows that false positives do exist, but the numbers are far lower than those obtained by mapping against plant species.

## CONCLUSION AND OUTLOOK

The upgrades described here provide miRNA researchers from all backgrounds with similar opportunities to analyze their sequencing data, provided that miRNA annotations of interest are available at all, rather than just focusing on annotations for model organisms or genomes that are of biomedical relevance. Furthermore, since we tend and intend to include more assemblies, miRNA and ncRNA annotations upon demand (as long as they meet certain quality criteria) we encourage potential users to request annotations of interest that they find missing. We welcome all kinds of feedback from the community which usually leads to interesting developments and improved features.

Future improvements include extended summarizing capabilities linked to the sRNAbench output page together with the analysis of RNA processing patterns and a full integration of artificial spike-in sequences.

## DATA AVAILABILITY

sRNAtoolbox webserver is freely available at https://arn.ugr.es/srnatoolbox/. Source code and executable files as well as instructions to start a docker with all the requirements can be found at https://github.com/bioinfoUGR/sRNAtoolbox.
